# The Enactive Approach to Architectural Experience: A Neurophysiological Perspective on Embodiment, Motivation, and Affordances

**DOI:** 10.3389/fpsyg.2016.00481

**Published:** 2016-03-31

**Authors:** Andrea Jelić, Gaetano Tieri, Federico De Matteis, Fabio Babiloni, Giovanni Vecchiato

**Affiliations:** ^1^Department of Architecture and Design, Sapienza University of RomeRome, Italy; ^2^Department of Psychology, Sapienza University of RomeRome, Italy; ^3^IRCCS Fondazione Santa LuciaRome, Italy; ^4^Department of Molecular Medicine, Sapienza University of RomeRome, Italy; ^5^Department of Physiology and Pharmacology, Sapienza University of RomeRome, Italy

**Keywords:** enactive approach, architectural experience, embodiment, body schema, emotion, motivation, affordances, virtual reality

## Abstract

Over the last few years, the efforts to reveal through neuroscientific lens the relations between the mind, body, and built environment have set a promising direction of using neuroscience for architecture. However, little has been achieved thus far in developing a systematic account that could be employed for interpreting current results and providing a consistent framework for subsequent scientific experimentation. In this context, the enactive perspective is proposed as a guide to studying architectural experience for two key reasons. Firstly, the enactive approach is specifically selected for its capacity to account for the profound connectedness of the organism and the world in an active and dynamic relationship, which is primarily shaped by the features of the body. Thus, particular emphasis is placed on the issues of embodiment and motivational factors as underlying constituents of the body-architecture interactions. Moreover, enactive understanding of the relational coupling between body schema and affordances of architectural spaces singles out the two-way bodily communication between architecture and its inhabitants, which can be also explored in immersive virtual reality settings. Secondly, enactivism has a strong foothold in phenomenological thinking that corresponds to the existing phenomenological discourse in architectural theory and qualitative design approaches. In this way, the enactive approach acknowledges the available common ground between neuroscience and architecture and thus allows a more accurate definition of investigative goals. Accordingly, the outlined model of architectural subject in enactive terms—that is, a model of a human being as embodied, enactive, and situated agent, is proposed as a basis of neuroscientific and phenomenological interpretation of architectural experience.

## Introduction

**Figure 1:**
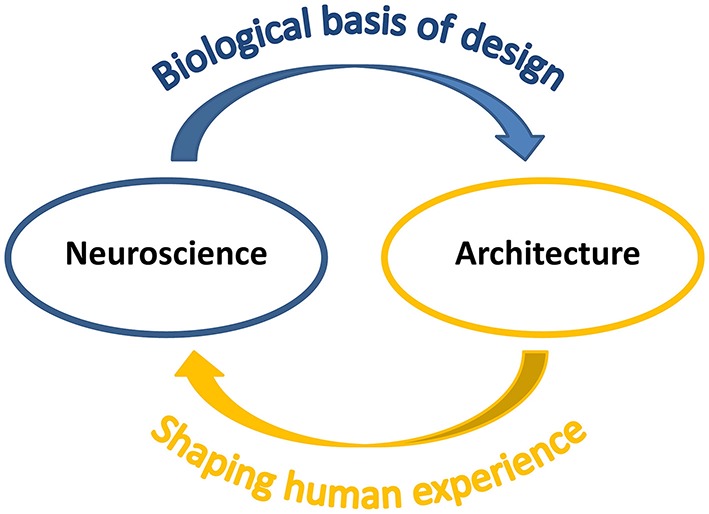
**Connection between neuroscience and architecture**. The study of the human being through neuroscientific methods provides quantitative measures and theoretical explanation for biological bases of design. At the same time, architecture influences human mind, physical well-being and behavior by shaping human experience.

The unique cultural position of architecture as an existential art—an art that scaffolds human life—has been openly advocated ever since the earliest architectural writings surviving from ancient times. Through the centuries, this architecture's double task of “showing and serving” (Leatherbarrow, 2009, p. 8) has been recognized as the value of architecture for human well-being. On the one hand, architecture enables the physical comfort of basic sheltering and everyday functionality, while having the capacity to emotionally move the human soul and nurture people's identities by articulating the wider cultural and societal conditions. As anticipated by Neutra (1954) in his definition of architects as gardeners of nervous growth, this relationship between architectural space and the human mind and body is acknowledged today through the explicit link of neuroscience and architecture. The intersection of the two disciplines explores the possibilities of shaping the human experience and well-being through a neurobiological approach to design (Sternberg and Wilson, 2006; Eberhard, 2009; Figure 1).

From historical perspective, architects' interest in human behavior is by no means a recent phenomenon—in fact, the field of architectural psychology has attracted continuous attention ever since Lynch's (1960) seminal study on imageability and wayfinding in urban environments. More recently, this ongoing environment-behavior research has been boosted with the novel hypotheses from neuroscientific discipline. As a result, current efforts have been following multiple lines of investigation—from the studies exploring connections between visual perception and spatial geometry to inquiries about embodiment and emotional impact of different environmental characteristics. Hence, for instance, one line of research investigates premises based on combined insights from information-processing approaches to visual perception (e.g., contour preferences, Bar and Neta, 2006) and classical architectural psychology theories (e.g., habitat theory (Appleton, 1975), informative environment theory, (Kaplan and Kaplan, 1989), and attention restoration theory, Kaplan, 1995). Such recent endeavors include: an exploration of neural correlates of restorative design elements as a way of providing the building occupants with the cognitive and affective resources necessary for adequate human functioning (Martínez-Soto et al., 2013), an examination of emotional responses to specific visual properties like contour in the context of architectural form and approach-avoidance decisions (Nanda et al., 2013; Vartanian et al., 2013), and a study of emotional reactions and preferences of architects and non-architects to various three-dimensional spatial geometries (Shemesh et al., 2015). To date, the limits of these approaches is reflected in the emphasis on interpreting architectural spaces in terms of objects with quantifiable properties, thus tackling only one aspect of a more complex atmospheric qualities of space (and not necessarily a decisive one for the overall architectural experience). Moreover, because the physical properties of spaces are quantified independently of perceiver as bodily subject, he/she is transformed into a disembodied observer.

Similarly, a number of studies have aimed at understanding the relations between visual perception, preferences, and configurational features by intertwining neuroscience and cognitive psychology with the Space Syntax methodology for spatial analysis^1^. The distinctiveness of space syntax methodology resides in the capacity to quantitatively map configurational and visual properties, such as building layout complexity. Although such spatial analysis have returned consistent results regarding the perceived spaciousness and orienting clarity of a particular environment (Dzebic et al., 2013), the capacity of this methodology to directly link emotional and aesthetic responses to other than configurational (topological) properties has been somewhat limited (Skorupka, 2010; Kuliga et al., 2013).

In parallel, there is an increasing interest for studying perception of architecture in terms of multi-sensory and embodied experience, understood in the context of late-nineteenth century empathy theories and inspired by recent conceptual and experimental neuroaesthetic framework by Freedberg and Gallese (2007). Accordingly, initial studies investigated how spatial features modulate bodily self-consciousness (Pasqualini et al., 2013) and explored the possibility to understand neurophysiological correlates of architectural perception pertaining to cerebral circuits involved in embodiment, sensorimotor integration, and spatial navigation (Vecchiato et al., 2015a,b). As elaborated later on, the proposed enactive approach for understanding architectural experience is directly based on this closely related line of research dedicated to the study of embodiment in art experience.

Taken together, the results of these initial efforts suggest that there is a possibility of developing a new neuroscientifically informed stance toward the human being as an architectural subject that can be endorsed in user-centered design. Most importantly, such results directly support the recent “experiential turn” among architects (i.e., the idea of human-centered design), proposed as a way to address some of the crucial contemporary architectural problems—such as the dominance of vision and intellectualization of designs manifested in the phenomenon of a disembodied architectural observer (Pallasmaa, 2005; Mallgrave, 2011). For this reason, it is important to note that although understanding of complex phenomenon such as architectural experience necessarily requires a well-thought fragmenting of research questions, any research perspective which is to yield results applicable to user-centered design should be formulated in accordance with the broader architectural discourse on experience (Jelić, 2015). Therefore, in order to replace the disembodied model of architecture user with the more accurate biological approximation of the human body as experiencing subject, there is a need to develop a systematic and coherent framework for theoretical and experimental investigations for this replacement to be constructively implemented in the design process.

In this context, the enactive approach is proposed as a guide to studying architectural experience for two key reasons. Firstly, the enactive account places emphasis on the situated nature of perceptual experience, which makes the issues of embodiment and relational embeddedness in the world vital to understanding people's engagement with architectural environments. This suggests that the way in which we perceive, experience, and engage with architecture depends on the particular kind of body we have and the possibilities for body-environment interactions that are inscribed in terms of the motor or skillful knowledge as potential for action. On the other hand, this posits a hypothesis of a two-way dependence: architecture is an expression of man's embodiment, while the way architecture is embodied influences the human mind, physical well-being, and behavior. The second incentive resides in the fact that enactivist perspective has a strong foothold in phenomenological thinking, which corresponds to the existing phenomenological background in architectural theory. Specifically, the enactive understanding of architectural experience corresponds to the phenomenological conception of architecture user as an embodied experiencing subject—as a body (capable of) moving in space resulting in enmeshed experience. As highlighted by numerous architects it is the body itself that acts as a measure of architectural quality (Zumthor, 1999; Holl et al., 2006). In this way, it is possible to identify and test hypotheses already present in architectural discourse and thus, to provide evidence-based grounding to architectural theories and design approaches that are commonly a thoughtful result of accumulated professional experience and yet, which are potentially dismissed as unconfirmed and speculative.

Therefore, as elucidated by Mallgrave in the *Architect's Brain* (Mallgrave, 2011), it can be proposed that one of the advantages of neuroscientific investigations of architectural experience is the possibility to verify a link between architect's intuitive understanding of phenomenal body and articulation of built spaces. In fact, by identifying hypotheses already present in architectural literature and in the form of designer's knowledge, it is possible to construct a strong frame of reference for comparing neuroscientific results with examples of well-designed spaces, like in the case of documented experiential quality of architectural masterpieces. Most importantly, it can be argued that the dialogue between neuroscience and architecture is less aimed at responding to scientific questions as it is a search for systematic structure underlying architect's design manipulations. For this reason, the common background based on the large body of knowledge from both neuroscience and architecture is proposed to be utilized through the enactive approach as a possible interpretation of architectural experience as a way of empowering architects to confront design tasks with an increased awareness of our biological and phenomenal nature.

We start from the premise that architecture can be described as a designed interaction between life and form. Accordingly, the enactive approach to architectural experience brings together the biological perspective on the human being through the concepts of embodiment and motivation on the one hand, and affordances as an artificially designed possibility for interaction, on the other. These ideas are developed in the following sections first by sketching the enactive approach to cognition, emotion and experience, and explaining perception as action-oriented. Then, emphasis is placed on the role of the body schema and bodily perspective in understanding architecture, as well as on the concept of affordances as design intentions in order to describe architectural experience as being fundamentally an interaction. Finally, we discuss virtual reality environments as practical and valuable tools to investigate perception and action in architectural spaces. These issues will be outlined from the phenomenological and neuroscientific points of view.

## The enactive approach to architectural experience

As phenomenologists like Merleau-Ponty (1962, p. 129, 1964) have firmly established, “the body is our general medium for having the world” and therefore, our mode of experiential access to the world of architecture. Accordingly, in order to investigate how people experience built spaces it is first necessary to rethink in enactive terms the nature of perception and the related phenomenological concept of the lived body. Particular emphasis is thus placed on the situated character of perceptual experience and the importance of our embodied manner of being in the world. In the following section we outline the key principles of the enactive approach for providing an interpretation of the architectural subject as experiencing and perceiving agent that can be of relevance for user-centered design.

### The enactive approach to cognition and experience: general framework

Varela et al. (1991) conception of cognition as enactive and embodied was primarily elaborated as a criticism and an alternative to the traditional cognitivist model of the mind as an information-processing device. In order to overcome the shortcomings of this disembodied cognitive model in which the mind and the world are considered as two pre-given and independent entities, Varela et al. emphasized the profound connectedness of the organism and its environment, accomplished in an active and dynamic relationship. Starting from this premise, they established the enactive approach, where the term *enaction* signifies a concept that a living being is an autonomous agent that actively generates and maintains its own cognitive domain through continuous reciprocal interactions of the brain, body, and the world (Varela et al., 1991; Thompson, 2007). Today, more than 20 years after the milestone work *The Embodied Mind* (Varela et al., 1991), there is an abundance of models and explanations that potentiate the coupling relation between the organism and environment and that are grouped together under the recently coined heading of the *enactive, embodied, embedded, extended*, and *affective*, i.e., *the 4EA approach to cognition* (Ward and Stapleton, 2012; Vörös, 2014). Although these variations currently do not amount to a unified theory of cognition (Menary, 2010; Shapiro, 2011; Di Paolo and Thompson, 2014), several attempts have recently been made to indicate that such possibility nevertheless exists. For instance, as argued by Ward and Stapleton (2012), if cognition is indeed enactive, then it is also embodied, embedded, affective and (possibly) extended. Their argument is based on the fundamental premise—first posed by Varela et al. (1991)—that perception and cognition essentially depend upon the organism's interactions with its environment. In other words, perception and cognition are constrained and shaped by conditions of embodiment of that cognizing bodily agent. In a similar fashion, Di Paolo and Thompson (2014) advocate that if embodied cognitive science is to offer a genuine alternative to more traditional cognitivist view, the notions of “body” and “cognition” and their relationship should be defined in accordance with the enactive account.

For these reasons, the enactive approach as it is here used to describe architectural experience is understood as a unified position which primarily follows the embodied-enactive view of the mind as envisaged by Varela et al. (1991) and Thompson (2007). In addition, emphasis is placed on the affective dimension of experience following the increasing amount of empirical evidence and theoretical models which highlight the fundamental role of the affective component in cognition and the perception-action cycle (Colombetti and Thompson, 2008; Bower and Gallagher, 2013). Importantly, this line of enactivist thinking is compatible with the understanding of the body and lived experience in architectural tradition based on the shared phenomenological interpretation of embodiment by Merleau-Ponty (1962, 1964).

**Figure 2:**
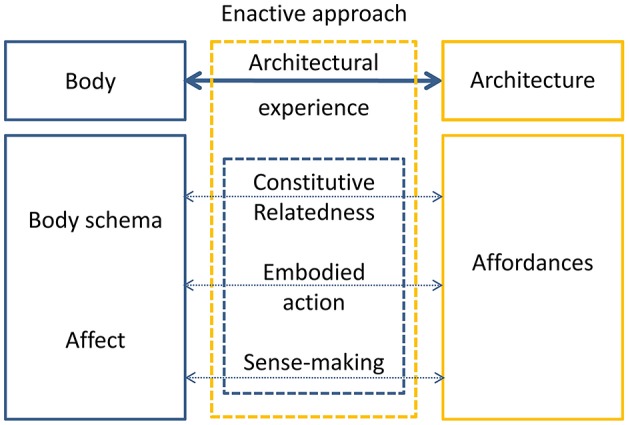
**The schematic account of enactive approach to architectural experience**. The enactive approach to architectural experience describes through the concepts of embodiment and affordances the interactive interface between body and architecture. Such interface is composed by three layers which dynamically participate in the architectural experience: the constitutive relatedness describes the living-lived body relationship between cognitive activity and experience; the embodied action defines cognition as bodily interaction with the environment through sensorimotor coupling of perception and action; the sense-making represents cognitive beings as self-individuating and sense-producing systems thanks to their bodily organization. These three aspects influence the human being through his/her body schemas and affective component, as well as architectural affordances.

Based on the description of key tenets of enactivism as provided by Thompson (2005, 2007) and with moderate simplification for the purposes of architectural discussion (Figure 2), it can be argued that there are three fundamental themes underlying the enactive approach: *sense-making, constitutive relatedness* (through embodiment), and *embodied action* (as sensorimotor coupling of perception and action). Originating in Merleau-Ponty's phenomenology and Varela's biological research, the idea of *sense-making* epitomizes the deep continuity between life and mind: what makes living organisms cognitive beings is their (bodily) organization as self-individuating and sense-producing systems (Thompson, 2007; Thompson and Stapleton, 2009; Di Paolo and Thompson, 2014). Firstly, living beings are considered to be autonomous agents that actively generate and sustain themselves and thereby instantiate their own cognitive domains. Secondly, they engage with the world in such manner that these interactions transform the world into a place of salience, meaning, and value (Thompson and Stapleton, 2009). As Colombetti (2014) argues, this is because all living systems have a fundamental “lack of indifference” to the world, which can be termed *primordial affectivity*. From the enactive viewpoint, emotion is accordingly understood as inherent constituent of the perception-action cycle, in such way that perception and emotion are treated as interdependent aspects of intentional action, which is thus always endogenous or generated from within (the organism) and is directed outward into the world (Thompson, 2007; Colombetti and Thompson, 2008). For this reason, the organism as a whole is understood as a vehicle of meaning because the significance and valence in the world is created by the organism itself, and therefore, sense-making can be more accurately described as a bodily cognitive-emotional form of understanding (Colombetti, 2010).

By being a vehicle of sense-making activity, the body thus has a fundamental role in constituting the way (human) beings enact and understand the world. In other words, whatever a being is able to experience, know, and practically handle is determined and shaped by the particular features of that being's body (Thompson, 2005, 2007). The crucial consequence of such understanding of the body and mind is the possibility of relating the objective body/brain, observed from (neuro)scientific perspective, and the perceiving and experiencing body, understood from the embodied first-person phenomenological perspective (Thompson, 2004). Therefore, by drawing from Merleau-Ponty's view of human existence as “doubly” embodied—on the one hand, as a living, objective body or a physical context of cognitive activity, and on the other, as a lived body or a bodily subject—this living-lived body relationship can be described in terms of the intrinsic *constitutive relatedness* between cognition, body, and the world. What this means in enactive terms is that a cognitive being's experiential world is “a relational domain enacted or brought forth by that being's autonomous agency and mode of coupling with the environment” (Thompson, 2007, p. 13). This fundamental relatedness is possible because cognition is defined as a form of *embodied action*—that is, an exercise of skillful know-how by an embodied and situationally embedded agent (Thompson, 2007). Cognition is thus conceived as constitutively dependent upon the conditions of embodiment and it is modulated by the way of sensorimotor patterns that are “exercised” through the bodily interactions with the environment. This line of reasoning is also evidenced in the particular structure of the human nervous system, which evolved as a link between movement and continuous stream of sensory activity. More specifically, this sensorimotor intertwining is organized in such way that “what the organism senses is a function of how it moves, and how it moves is a function of what it senses” (Thompson and Varela, 2001, p. 424). Therefore, it can be argued that the perception-action cycle is crucial for enabling the organism to be a situated cognizing agent—that is, to exist in a meaningful relation with the environment—and which thus emphasizes the role of the body as a “vehicle for being-in-the-world” (Merleau-Ponty, 1962).

The enactive approach considers perception as “an embodied coping with the environment” (Gallagher and Zahavi, 2008, p. 99) and therefore, as essential to the organism's manner of being and knowing the world. Accordingly, grasping perceptually the world and things in space presupposes the existence of egocentric spatial frame of reference which can be acquired through spatial relations between perceived objects and the body. These are always defined by virtue of the orientation they have to our perceiving and acting (i.e., moving) bodies (Merleau-Ponty, 1962; Thompson, 2007; Gallagher and Zahavi, 2008). From phenomenological point of view, the body functions as “an absolute indexical ‘here”’ (Thompson, 2007, p. 248) or a “degree zero of spatiality” (Merleau-Ponty, 1964, p. 178), meaning that the space a person inhabits is constituted in regard to the referencing zero-point which is always a lived, perceiving body. Because the world is perceiver-dependent, our existence can be described as inherently spatial, where this spatiality is an expression of the sensorimotor coupling between the organism and its environment (including the built environment). Hence, this co-constitution of the perceiving agent and the world (or more specifically, architecture) can be made apparent by understanding the modes of embodiment of the human being as a living-lived body, which requires a rigorous scientific and phenomenological analysis, as envisaged by the enactive approach.

### Sensorimotor theory of perception and experience of architecture

Alongside the enactive approach as a broad framework for studying cognition, there is a more focused line of investigation dedicated specifically to the enactive theory of perception, better known as the *sensorimotor (contingency) approach*. This field of inquiry was initially launched by O'Regan and Noë (2001) with their proposal for the sensorimotor account of visual perception. In their words, visual experience is the activity of exploring the world which is mediated by the knowledge of sensorimotor contingencies—that is, by understanding implicitly the regularities in which sensory stimulation changes with movement (O'Regan and Noë, 2001; O'Regan et al., 2005). The novelty of this view lies in the shift away from the “passive snapshot” toward “active exploration” concept of vision, which emphasized the embodied-enactive understanding of the mind and (conscious) experience as brought forth by the agent's way of interacting with the world (Bishop and Martin, 2014). In short, perceiving is understood as a way of acting: perception is not something that happens to us, or occurs inside us, but it is something we do (Noë, 2004). In addition, the important upshot of Noë and O'Regan's sensorimotor theory is the understanding of differences between modalities of perceptual experience, i.e., seeing, hearing, touching, and so on, as originating in laws of sensorimotor dependencies that are unique for each sensory system. The sensorimotor patterns depend on *sensory* contingencies—that is, the particular characteristics of the sensory apparatus (e.g., sense anatomy), as well as the features of the world to which the apparatus is sensitive (e.g., light, odor, sound waves etc.)—and on the other hand, on *motor* contingencies that differentiate sensory experiences by virtue of responding to particular movements (e.g., eye, head, or other bodily movements) (O'Regan and Noë, 2001; Shapiro, 2011).

In the spirit of the enactive approach, it is significant to ward off some of the typical concerns regarding the nature of sensorimotor knowledge. Specifically, the ability to know what is being perceived (e.g., what shapes I am looking at) consists in the practical mastery of the regularities governing the ways of exploring the world (e.g., the shape is given experientially as a sensorimotor pattern). This mastery of the sensorimotor rules is not a kind of propositional, explicit knowledge, but the perceiver's implicit know-how of the sensorimotor dependencies between one's sensing and moving body and the environment (O'Regan and Noë, 2001; Noë, 2004; Beaton, 2013). Underpinning this skillful mastery is the certain kind of “perceptual attunement” of the organism to arising sensorimotor patterns, which is ultimately grounded in the organism's embodied form and structure. Importantly, this perceptual attunement, i.e., particular sensitivity for the ways sensory stimuli change with movement (Myin and Degenaar, 2014), is the result of an embodied history of interactions, where the appropriate action is discriminated on the basis of past experience and in accordance with current goals (Buhrmann et al., 2013). Hence, attunement does not result in representational knowledge but in refined attunement.

More recently, other scholars of enactivism have argued that O'Regan and Noë's sensorimotor contingencies approach supports a narrow view of embodiment in terms of neuro-muscular function, while neglecting the motor intentionality of the bodily agent (Thompson, 2005; Buhrmann et al., 2013) and the influence of other bodily factors like affective states (Gallagher and Bower, 2014; Scarinzi, 2014). They argued that for the sensorimotor account to be in accordance with the enactive approach to perception and cognition it should embrace a richer phenomenological sense of embodiment reflecting an experiencing, bodily subject. For instance, Bower and Gallagher (2013) emphasized that our perceptual openness to the world (i.e., what we can perceive at any instance in time) depends not only on the mastery of sensorimotor contingencies, but also on our preconscious affective, motivational states that are intrinsic component of action-perception cycles. As described in the previous section, understanding the sensorimotor theory of perception from this enriched 4EA perspective is essential for the specific enactive approach to architecture elaborated here. Moreover, recent evidence from virtual reality studies investigating the relationship between perception, motivational factors, and neurophysiological mechanisms of embodiment support such a unified view. Section Immersive Virtual Reality as a Tool for Neuroscientific Investigation of Architectural Experience proposes how enactive approach could be explored in the VR setting.

Interestingly, a strong similarity can be observed between the embodied-enactive approach with its emphasis on perception, action, emotion, and cognition as dynamically intertwined and the argument that basic architectural experiences have a verb form^2^ because architecture initiates, directs, and organizes behavior and movement (Pallasmaa, 2005, 2011). For Pallasmaa, authentic experiential or mental constituents of architecture are always “*confrontations, encounters*, and *acts* which project and articulate specific embodied and existential meanings” (Pallasmaa, 2011, p. 124). In the light of sensorimotor theory, architectural space might be a prototypal example of our embodied nature and perceptual attunement to the environment. In particular, in our encounters with architecture, people do not have to think consciously about how to maneuver their bodies through a doorway or up the staircase because they are already in possession of an implicit sensorimotor knowledge that enables them to grasp spatial relations in a practical, pre-reflective manner. Ever since early childhood's explorations of different architectural spaces, people are fine-tuning the skillful mastery that allows them to experience each new place. Most importantly, it is precisely because we have a profound bodily memory of an intimate, human-scale architecture, when confronted with a less successful design we can indeed feel the discrepancy between what is perceived and how we might (or not) inhabit that space with the bodies we have. Therefore, enactive investigations of architectural experience could reveal how architectural space is shaped by the laws of human embodiment. At the same time, architecture's evident capacity to engage our sensorimotor systems could provide new knowledge on the human perceptual and cognitive functions and the underlying neural activity.

## The body as a communicative point for architectural experience

### The role of body schema in architectural experience

Speaking in enactive terms, the intrinsic constitutive coupling between perception, action, and emotion is fundamental for capturing our reality as lived bodies and experiencing subjects, and at the level of the organism as a whole is organized as the functional mechanism of body schema. The body schema is a concept extensively used in a variety of disciplines, including neuroscience and psychology, to describe one's capacity to act coherently in the world and be aware of one's own body (Berlucchi and Aglioti, 2010). Developed along the same lines, this concept is closely related to the phenomenological understanding of the lived body as a living and feeling agent, where the body schema is directly involved in the pre-reflective bodily self-awareness (Merleau-Ponty, 1962). From the enactivist perspective, these two views—the neuroscientific and the phenomenological—are taken together and are translated into two crucial characteristics of body schema. On the one hand, it is a system of largely prenoetic and close-to-automatic processes that constantly regulate posture and movement; at the same time, body schema plays a fundamental role in providing us with a minimal sense of self—that is, with a pre-reflective consciousness of ourselves as experiencing, lived bodies (Gallagher, 2005; Gallagher and Zahavi, 2008; Berlucchi and Aglioti, 2010). For that reason, body schema is of particular interest for the enactive interpretation of embodiment, since it brings together the personal and subpersonal, i.e., the first- and third-person perspective. Accordingly, we propose that body schema lies at the essence of *pre-reflective architecture-body communication* for two reasons: firstly, because it enables us to engage with the built environment in a profoundly animated, pragmatic, and meaningful manner, and secondly, because it provides us with an access to our bodily self and thus, to conscious experience of a situation (e.g., architectural space) at hand.

It is worth noting that because of its long history, the term body schema has often been used interchangeably with body image. However, these concepts are not equivalent, although they are tightly connected and necessary to capture the complexity of the human mind (Gallagher, 2005). The body image and body schema are phenomenologically differentiated in such manner that the first implies taking or having an intentional (objective) attitude toward one's own body, while the other signifies the capacity to move and exist in the bodily action. Thus, the body image is a sometimes conscious system of experiences, attitudes, and beliefs pertaining to one's own body, where this understanding of the body as an object can be also influenced by cultural and scientific knowledge and interpersonal factors (Gallagher, 2005; Gallagher and Zahavi, 2008). Accordingly, it is the investigation of the body schema that allows us to delve into the manner of individual's active engagement with the (architectural) environment.

Body schema can be defined as a continuously updating neural representation of the body's configuration, which enables, monitors, and controls the body shape and posture, as well as its position and movement in space. These bodily representations are used to compute not only the position, shape and dimension of the target of our actions, but also of our own body and, in particular, of the body-part we want to use to execute the action. Body schema functions as a set of unconscious and tacit performances which combine and synchronize bodily information coming from somatosensory modalities, such as proprioception, kinesthesia, and touch, into a sensory-motor schema (Cardinali et al., 2009). Significantly, body schema includes not only the processes underlying motor activity and the regulation of postural and kinesthetic information, but also the interoceptive emotional inputs as motivational propensity for action (Berlucchi and Aglioti, 2010; Bower and Gallagher, 2013). For that reason, as Bower and Gallagher (2013); Gallagher and Bower (2014) have recently emphasized, body schema gives an agent the “how” of perception through the tacit knowledge of potential sensorimotor engagements with the environment, while the “why” of perception depends on the latent motivations that direct one's actions and attention (more details on the role of affect and latent motivations will be discussed in Section Affective Components of Perception).

Accordingly, body schema can be understood as the sensory-motor representation of the agent's body and its action possibilities—it functions as a set of dynamic sensorimotor principles that organize perception and action. As such, its role pertains to motor control, both voluntary and autonomic, and to the kind of skillful knowledge emphasized by the enactive theory of perception. Moreover, in accordance with phenomenological views by Merleau-Ponty, our primary way of being in the world is in a bodily and skillful manner, since our body schema is defined as a vehicle of one's bodily or motor intentionality (Merleau-Ponty, 1962). Our hold on the world is primarily the one of a pragmatic intentional action, with the lived body manifesting in perceptual experience as an “implicit and practical ‘I can’ of movement and motor intentionality” (Thompson, 2005, p. 411). Accordingly, what means to be an experiencing bodily subject is to be “an agentive body that moves in action” (Gallagher, 2014, p. 10), implying that body schema not only monitors bodily states but that it is fundamentally action-oriented (Gallese and Sinigaglia, 2010). On such grounds, it has been suggested that the pre-reflective bodily self-consciousness consists in “experiencing one's body as the point of convergence of perception and action” (Legrand, 2006, p. 108) and thus conceiving the bodily self as “an integrated system characterized by matching of sensory-motor information” (Legrand, 2006, p. 111). What is significant about this view is that it corresponds to the embodied-enactive approach to cognition, suggesting that the self appears from the interaction of the organism with the environment. Closely tied to both Legrand's and enactivist viewpoints is the argument by Gallese and Sinigaglia (2010) who proposed that the sense of the body as an experiencing body (i.e., sense of self) is primarily given to us as the “source” or “power” for action, based on its intentional character and the variety of motor potentialities available to the body through body schema. Therefore, the bodily self defined as power-for-action essentially presupposes the sense of ownership of an action-capable bodily agent and as such, its functionality primarily rests on the capacities of the motor system (Gallese and Cuccio, 2015). As a matter of fact, recent evidence has been corroborating this hypothesis that the bodily self might be rooted in the complex brain systems that represent the body (Ferri et al., 2011) and have revealed a sensorimotor neural network for the general representation of the bodily self (Ferri et al., 2012).

**Figure 3:**
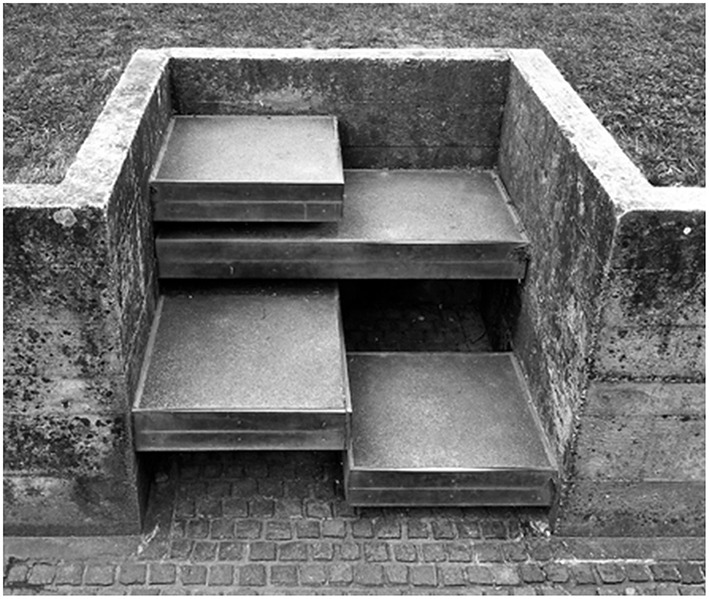
**Architectural spaces can act directly on attention and conscious awareness through body schema by disrupting the habitual engagement with space**. Example of peculiar Carlo Scarpa's stairs at the Brion Cemetery in San Vito d'Altivole, Italy which requires the visitor to recalculate the body's position and appropriate action. This brief instance is just enough to activate the attentional switch and to allow the visitor to consciously experience both the architectural settings and oneself as an experiencing and bodily subject.

At the same time, the capacity of body schema to switch the conscious attention to the body itself (e.g., like when passing between tightly parked cars) has been successfully employed by architects throughout history to immerse the perceiver into a spatial situation. For instance, this “rupture” of the habitual, pre-reflective use of space by acting directly on body schema is applied in the design of Carlo Scarpa's peculiar stairs at the Brion Cemetery in San Vito d'Altivole, Italy, where each step is dedicated to stepping with either left or right foot (Figure 3). Here the moment needed to recalculate the body's position and appropriate action is just enough to activate the attentional switch and to allow the visitor to consciously experience both the architectural setting and oneself as an experiencing and bodily subject. It is in this sense that body schema can be recognized as having an essential role in architectural experience and why architects could benefit from an elaborate enactive interpretation of this bodily mechanism. Specifically, it can be proposed that such conception of body schema can guide future studies addressing the issue of aesthetic stance, as well as the experiential and perceptual differences between the conscious and unconscious attitudes toward architecture. Thus, by taking distinguished examples from rich architectural history as investigative settings, it might be possible to analyze how spatial structures can act as attentional cues through the mechanism of body schema. Such understanding would aim to include not only visual cues, but to emphasize the effects of embodied architectural cues—the ones which stem from proprioceptive and tactile stimuli as a result of individual's interaction with the environment.

### Neuroscientific evidence of action-perception and empathy in aesthetic experience

Almost a decade ago, Freedberg and Gallese (2007) proposed a theoretical framework for studying aesthetic experience based on the neuroscientific interpretation of the theory of empathy as a corporeal and emotional resonance with an artwork. Their starting ground was the notion of empathy as *Einfühlung* in its original sense of the bodily experience of art and architecture, as developed in the late-nineteenth (Robert Vischer, Heinrich Wölfflin) and early twentieth (Theodor Lipps) century psychological aesthetics. For Vischer, the concept of *Einfühlung* (literally “feeling-into”) signified the unconscious projection of our “own bodily form—and with this also the soul—into the form of the object” (Vischer, 1994, p. 92). By embracing the embodied simulation theory of empathy, Freedberg and Gallese (2007) offered an interpretation of aesthetic experience as involving the motor system and the related activation of embodied mechanisms. Importantly, following Wölfflin's study on empathy in architecture (Wölfflin, 1994), they suggested that these processes could be involved in the perception of architectural spaces as well as visual arts. In a subsequent meta-analysis, Brown et al. (2011) argued that aesthetic processing can be considered as the appraisal of valence of perceived objects and moreover, that it could be a general process applicable to both art and non-art objects, and involving cerebral areas serving biological and social needs.

The main hypothesis of Freedberg and Gallese's approach is the involvement of the motor system in aesthetic experience, where embodied simulations are described as empathy for tactile sensations, implied gestures and actions. Thus, the observer is automatically able to create an empathic feeling with the representational content of the artwork or for example, with the artist's explicit gestures (Di Dio and Gallese, 2009). Since then, several studies demonstrated the activation of motor circuits during the perception of artworks. Di Dio et al. (2007) showed that the premotor cortex and the inferior parietal lobe are activated during the observation of images depicting classical sculptures, which evoked a sense of action in the observer due to the implicit dynamic properties of the sculpted human figures. The sensorimotor area was also involved in beauty judgment, specifically in the case of the negative aesthetic evaluation of modified stimuli (i.e., the original sculpture with altered body proportions). In this case, based on the simultaneous decreased activity in orbitofrontal cortex, authors proposed that the motor activation in response to ugly stimuli is a covert release of a motor behavior. Umiltà et al. (2012) reported that the observation of images depicting original abstract artworks, such as Lucio Fontana's cut canvases, elicited a mu rhythm suppression across bilateral motor areas. Authors discussed that such cerebral activations were engendered by the presence of cuts on the canvas as static representation of motor acts (i.e., a consequence of the artist's intentional hand gesture). In addition, the modulation of the mu rhythm did not depend on the familiarity of the visual stimuli. Similarly, a group of researchers investigated if a mu suppression could be elicited by the observation of Rorschach cards (Pineda et al., 2011). They related the 8–13 Hz frequency band suppression at scalp central sites to the internal representation of the feeling of movement elicited by inkblots.

In a following study, Sbriscia-Fioretti et al. (2013) used reproductions of Franz Kline's abstract paintings to assess in detail the involvement of sensorimotor circuits during the observation of static consequences of hand gestures (i.e., brushstrokes). By means of an event-related potential (ERP) high resolution EEG study, authors showed that the observation of original paintings generated an increase of cortical activity when compared with the observation of modified stimuli (i.e., computer generated “gestures”). Specifically, authors reported negative ERP deflections located at frontal and central scalp sites in the first 350 ms of stimulation. Most interestingly, the higher spatial resolution of the used technique allowed them to distinguish four cortical circuits activated in the perception of such artwork—networks which lie across the sensorimotor, visual, prefrontal, and orbitofrontal areas. As discussed, the activity of the sensorimotor cortex mediates a pre-reflective and automatic understanding of (implicit) actions in accordance with the embodied simulation theory (Gallese, 2005). Moreover, visual areas are known to be involved in processing of beauty and in particular, they could reflect the neural response to visual stimuli implying motion (Di Dio et al., 2007; Thakral et al., 2012). Finally, the activation of orbitofrontal cortex is associated with processing of pleasant and rewarding stimuli (Kawabata and Zeki, 2004; Vartanian and Goel, 2004; Lacey et al., 2011), and the enhancement of activity related to prefrontal areas is connected to judgment tasks of aesthetic parameters (Cela-Conde et al., 2004, 2011; Jacobsen et al., 2006).

Furthermore, important evidence for the role of emotion and reward in aesthetic perception of architecture comes from two recent studies on aesthetic judgments and approach-avoidance decisions in architectural spaces (Vartanian et al., 2013, 2015). Specifically, Vartanian and colleagues investigated the effects of curvilinear/rectangular contours (Vartanian et al., 2013) and ceiling height with perceived enclosure (Vartanian et al., 2015) on judgments of beauty and enter/exit decisions by projecting images of architectural spaces in a fMRI experiment. A distinctive response observed in both studies is the activation of medial orbitofrontal and anterior cingulate cortices, which is consistent with their established involvement in the core circuit for aesthetic processing (see Brown et al., 2011, and this section). In the first instance, judging the curvilinear spaces as beautiful and pleasant was associated exclusively with increased activity of the ACC (in contrast to spaces with rectilinear contours), while the second study showed greater activity in the anterior midcingulate cortex in the case of enclosed spaces, which elicited more avoidance judgments overall in comparison to open spaces.

Overall, these results provide evidence that aesthetic perception depends on the implicit and internal actions engendered by artworks, also including emotional experience and evaluation processing, as well as context related factors. This complex neuroscientific picture can be analyzed conveying these findings into three distinct but dynamically interconnected neural systems accounting for aesthetic appreciation. Specifically, these cerebral networks involve the sensory-motor, emotion-evaluation and meaning-knowledge systems which interact during object perception and are hypothesized to play an important role for appreciation of architecture (Freedberg and Gallese, 2007; Di Dio and Gallese, 2009; Chatterjee and Vartanian, 2014). According to this framework, the sensory-motor system automatically processes objects and features of the environment which engage the observer through embodied mechanisms; the emotion-evaluation system processes information related to approach/withdrawal as well as to wanting and liking, whereas the meaning-knowledge system is so far the least understood since it is widely distributed in the brain and strongly dependent on cultural contexts and individual expertise.

While the fine dynamic interconnection among these cerebral networks still remains to be investigated in detail, it is worth proposing a broader hypothesis on the nature of architectural experience (including its aesthetic dimension) and how these experimental results could correspond with the recent enactive accounts of aesthetic experience (Xenakis and Arnellos, 2014, 2015). Firstly, the evidence of the sensory-motor system in object perception suggests that aesthetic experience arises as a result of the interaction between the observer and the object. On the other hand, the role of emotion-evaluation systems indicates that aesthetic experience is an embodied phenomenon directly linked to adaptivity (Xenakis and Arnellos, 2015) and that aesthetic perception functions in the service of a better coping with the environment (Xenakis and Arnellos, 2014). Following such description of aesthetics, empathy as a bodily understanding of the work of art or architecture can be thus identified as pertaining to the process of sense-making and anticipatory preparation for subsequent actions.

### Affective components of perception

In the previous section we discussed recent findings showing that the observation of aesthetic stimuli involves cerebral areas forming the motor system and devoted to action perception. However, such neural activity cannot appear alone, as it is also hypothesized by the enactive approach. For instance, the aforementioned works showed that viewing an artwork activates inferior parietal lobule and the premotor cortex as manifestation of an embodied simulation, but also several areas which are involved in emotion processing, including deep nuclei (the anterior insula and amygdala), cortical regions (the orbitofrontal, anterior cingulate cortices, and the left prefrontal areas) (Di Dio et al., 2007; Sbriscia-Fioretti et al., 2013). Moreover, a recent meta-analysis also confirmed that besides the activation pattern of central areas processing action observation and execution (i.e., inferior frontal gyrus, ventral premotor cortex, and inferior parietal lobule), additional areas, such as cerebellum and the limbic system, are recruited. According to this study, these regions are thought to be responsible for integrating affective components of action (Molenberghs et al., 2012).

For instance, the insula represents viscerotopic map of ascending viscerosensory inputs from the body and regulate negative affective experience in general (Craig, 2009). This cerebral region is usually divided in anterior and posterior portions. Anterior regions are primarily associated with interoceptive awareness of the body and involved in motivational and affective states that have a strong visceral component (e.g., disgust), while posterior regions are connected with primary representations of sensations from the body (Critchley et al., 2004; Ochsner et al., 2012). Specifically, the anterior insula is anatomically connected with limbic structures and with centers involved in autonomic functions (Dupont et al., 2003), whereas from the functional point of view the insula mediates feelings related with emotional states (Damasio et al., 2000; Di Dio et al., 2007). Recent evidence indicated that interoceptive information about bodily states—both bodily and emotional feelings—is represented in the insular cortex, which is thought to be crucial for emotional experiences and conscious awareness of the environment and the self (Craig, 2009; Damasio and Carvalho, 2013). Correspondingly, recent accounts of body schema (Berlucchi and Aglioti, 2010) suggest that interoceptive signals, along with proprioceptive and exteroceptive information, are important for complete “tracing” of one's own bodily states and body's position in space. The amygdala is a complex structure which is anatomically connected with several cortical and subcortical regions. Functionally, it is involved in the perception and encoding of stimuli relevant to affective goals (Cunningham et al., 2008; Ochsner et al., 2012). One of the primary roles of the amygdala is to provide affective value to neutral stimuli through association learning; this nucleus could thus be responsible for storing and accessing emotional memories (Paton et al., 2006; Di Dio et al., 2007).

Along with the discussed cerebral nuclei, several cortical frontal regions are also involved in the emotional processing of aesthetic perception as part of the reward circuitry. In particular, the orbitofrontal cortex plays the role of signaling the actual value of a given stimulus, which varies according to internal preferences and judgments, independently of sensory modality. This cortical region is crucial when information about a specific outcome is necessary to instantiate a behavior and for learning (Schoenbaum et al., 2011). In addition, it also plays a role in sensory integration and decision making, and more recently, it has been proposed to mediate the hedonic experience (Kringelbach, 2005). Two recent meta-analyses also highlighted the role of the reward circuitry in the processing of aesthetic perception (Brown et al., 2011; Kühn and Gallinat, 2012). The orbitofrontal and the anterior cingulate cortices have a specific role in this processing. Specifically, the former works as a higher-level sensory cortex receiving input from sensory pathways involved in object processing (Rolls, 2005), while the latter predicts and monitors outcomes in relation to motivational intentions (Carter and van Veen, 2007). Moreover, Sbriscia-Fioretti et al. (2013) depicted a clear role of left prefrontal areas during the observation of abstract paintings in their experiment. These regions usually serve in judgments tasks but were also observed to be active in response to visual artworks (Cela-Conde et al., 2004, 2011; Jacobsen et al., 2006).

Davidson and colleagues described that different sectors of the prefrontal cortex are primarily involved in emotion and motivation processing along with the amygdala, hippocampus, insula and anterior cingulate cortex (Davidson, 2003). Each of these structures plays a different, complementary role in specific features of emotion. In addition, a lateralization of such activity could be also a sign of supporting theories related to functional (Davidson, 2004) and neuroanatomical (Craig, 2005) frontal asymmetries describing approach/avoidance behaviors. Specifically, the left prefrontal cortex plays a role in moderating patterns of activity related to approaching stimuli, whereas the right hemisphere of the frontal lobe is asymmetrically activated by avoiding stimuli. It is worth noticing that the prefrontal cortex is anatomically and functionally heterogeneous although there is extensive intrinsic connectivity among its various subregions. This pattern of anatomical connectivity can therefore provide the basis for orbital, ventromedial, and ventrolateral sectors of the prefrontal cortex to modulate processing in the dorsolateral sector. As to the origin of such cerebral frontal asymmetry, it seems there are genetic influences on EEG measures of prefrontal asymmetry, but at the same time, it has been suggested that environmental influences, particularly early in development, are likely to be present and to shape aspects of functional prefrontal asymmetry (Davidson, 2004). Moreover, Craig (2005) binds such psychophysiological evidence of cerebral frontal asymmetries to neurobiological roots. Hence, the asymmetric activity of the frontal lobe could be interpreted as epiphenomenon of the asymmetrical representation of homeostatic activity, originating from anatomical and functional asymmetries in the peripheral autonomic nervous system and connected with insular cortex and forebrain cardiac control (Craig, 2009). In this view, emotions appear to be organized for the management of physical and mental energy.

In this regard, there is an interesting hypothesis developed by Barrett and Bar (2009) according to which object perception does not only depend on previously encountered multisensory patterns but also on affective representations, i.e., prior bodily experiences that influenced the internal sensations. Although they do not speak explicitly about embodiment but rather about the “gist” which could arise at both conscious and unconscious levels, bodily sensations are understood as having a “dimension of knowledge” which helps to identify objects and actions according to past bodily reactions. This model relies on the functional connections between the visual areas and the orbitofrontal cortex which uses visual information to modify the perceiver's body state to re-create the affective context in which the object was experienced in the past. This neural process could participate in object perception and mediate following behaviors.

## Affordances enacted: experience of architecture as designed for action

### Affordances in architectural terms

In design fields like architecture and product design, the notion of affordances has been typically used to describe the functionality of designed artifacts in terms of the perceived usability by the user (e.g., the mobility and ergonomic properties of architectural elements and spaces). More recently, a clearer link has been established between designed affordances as action possibilities that can also invite behavior and the agent's capacities to perceive and engage with them (Withagen et al., 2012). However, so far results have been limited in attempting to describe this relationship in terms of the biological nature of an architectural subject. In contrast, as discussed in previous sections, the enactive approach to architectural experience makes it possible to hypothesize about the mutual impacts that architectural and human embodiment have on each other, and the extent to which architecture shapes our everyday life and culture as a whole. Therefore, in order to make more explicit the constitutive reciprocal relationship between the conditions of human embodiment and architectural space, it is purposeful to cast the notion of affordances into more enactive terms.

A notion first developed by Gibson (1986), affordances are defined as possibilities for action which are provided to an animal by its environment, including substances, surfaces, objects, and other living creatures that surround it. Over the last decades, the concept of affordances has been refined by many authors by placing explicit accent on the complementarity of the animal and the environment—that is, affordances are conceived as relations between abilities of animals and features of the environment (Chemero, 2003; Rietveld and Kiverstein, 2014; Xenakis and Arnellos, 2014). Defined in such way, affordances clearly resonate with the enactive view that the world and the organism are co-constituted because there is a viable coupling between what the world affords and our perceptual and practical capacities. On the one hand, the world (through affordances) informs what we can see and do, while at the same time, our perceptual abilities and capacities for skillful action play a role in demarcating—thus, perceiving and potentially engaging—with what is in our world (Ward and Stapleton, 2012). Therefore, by taking into account the enactive understanding of perception as a preparatory-anticipatory process, affordances can be defined as value-rich potentialities of interaction that emerge in agent's perception (Xenakis and Arnellos, 2013, 2014). It is important to note, however, that not all affordances are available to each organism or each animal species. Based on the Gibsonian concept of the primacy of the ecological niche, the landscape of affordances (Rietveld and Kiverstein, 2014) is a refined concept meant to signify that from an immense range of existing affordances, those that are available to a particular form of life (e.g., human beings) are constrained by two reasons: (i) by virtue of organism's embodiment, as it is also posited by enactivists and (ii) by the whole spectrum of abilities available in human socio-cultural practices. A subgroup of this landscape is a field of affordances which is defined to suggest that affordances are relations between aspects of the environment and available abilities of the individuals, where specific affordances are available to a particular individual at any moment in time depending on his/hers abilities, needs, and preferences (Rietveld and Kiverstein, 2014).

**Figure 4:**
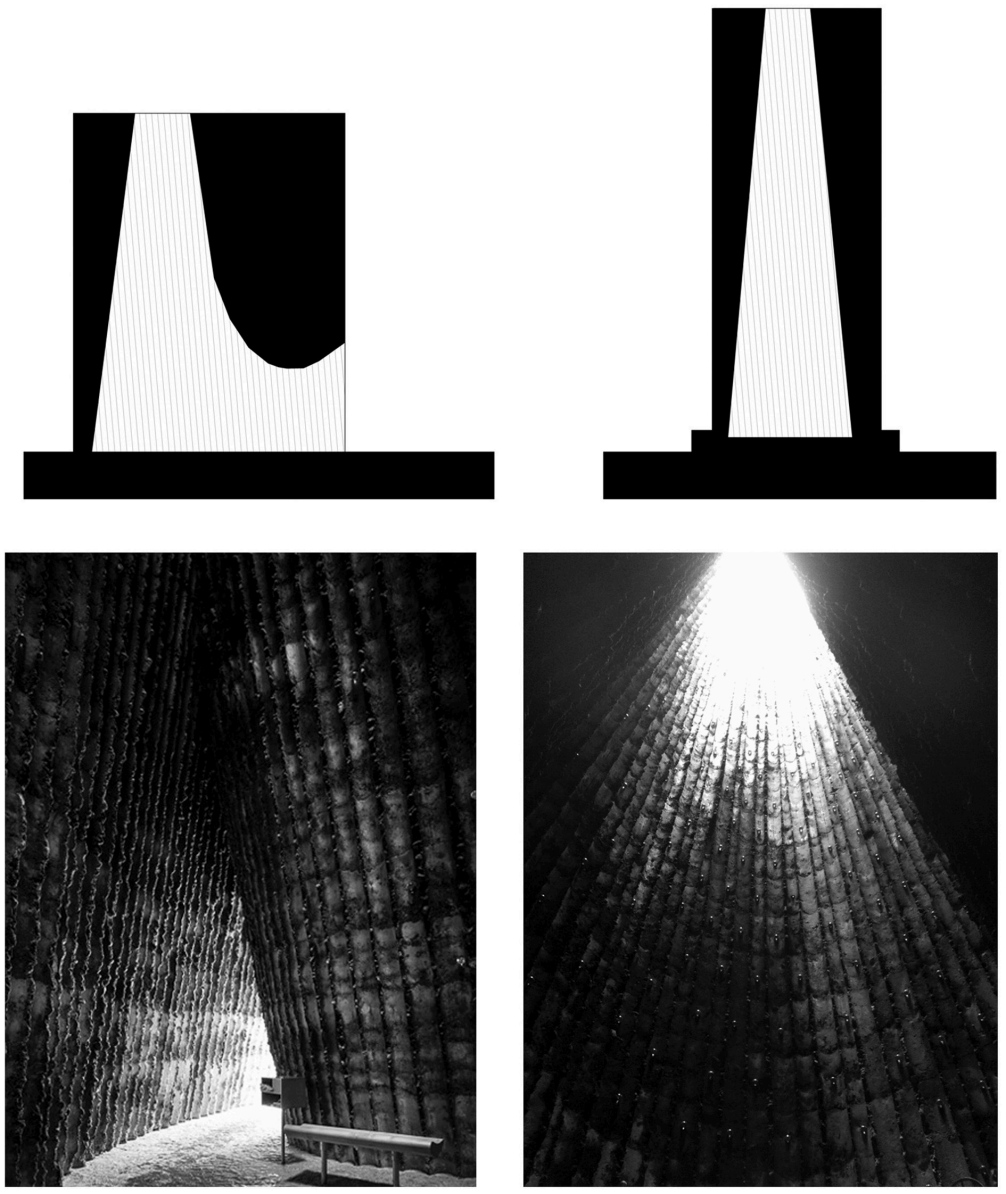
**Co-dependency between architectural and human embodiment**. Peter Zumthor's Bruder Klaus Field Chapel provides an example of architect's intentions expressed as an experiential scenario or a spatial articulation of affordances. Here, the experience of architectural space if directly structured on the basis of interaction between our embodiment, controlled through body schema, and provided (designed) spatial affordances.

Therefore, starting from the premise that architects design affordances, it might be suggested that people's experience of architectural environments is intrinsically structured by the possibilities for action, which is informed from both sensorimotor knowledge and motivational factors of every individual. More specifically, on account of presented concepts, a hypothesis can be put forward that the communicative space of architecture-body might be described as the conceptual coupling of body schema and affordances as “enacted” by architectural environments. A clear illustration of this point from architectural perspective can be found in the widely praised Peter Zumthor's Bruder Klaus Field Chapel in Mechernich, Germany. Although a small-scale intervention, this chapel demonstrates through simple conceptual and spatial intention the co-dependency between the architectural and human embodiment. As presented in Figure 4, the visitor's spatial experience includes two distinct bodily positions: starting from the unusual entrance through a dark passage with its narrowness being directly felt as bodily feelings (Pasqualini et al., 2013) toward the central space which expands vertically and requires the changes of posture with the look upwards, hypothesized to be in connection with the sense of awe (Eberhard, 2009). In this instance, the spatial structure is a direct expression of architect's intentions—moreover, it is an embodied or “spatialized” experiential scenario which presents itself to the subject as a scenario of affordances. Importantly, the effectiveness of selected affordances (narrow entrance to vertical central space) resides in the essential features of man's embodiment (upright posture and human scale). Thus, the experience of architectural space is directly structured on the basis of interaction between our embodiment, controlled through body schema, and provided, designed spatial affordances. Although Zumthor's work does not fall into a category of everyday architectural experiences, such extraordinary masterpieces are helpful as initial testing ground based on the clarity of architect's intentions, which in all well-designed spaces have to remain in the background of common life and work activities.

### Object perception and affordances

According to phenomenological works by a number of notable philosophers, for the lived, experiencing subject body schema provides embodied capabilities for action that correlate with the affordances of the world (Gallagher and Zahavi, 2008). For instance, a significant aspect of the Heideggerian account suggests that our primary way of existing is essentially a pragmatic action-oriented encounter with our environment (Moran, 2000). This practical and embodied character of our lived experiences implies that the manner in which things are presented to us is determined by the nature of the body as well as by our interests and goals; things are known through their “manipulability” or being “ready-to-hand” (Gallagher and Zahavi, 2008; Mallgrave, 2013). This concept of readiness, together with Husserl's notion of intentionality as “I can,” has found strong expression in Merleau-Ponty's phenomenology of embodiment, and more specifically in the concept of body schema as our “primordial grip” on the world, which is always a practical and intentional one, as described above. The body schema as a set of sensorimotor performances regulating the perception-action coherence and including the pre-reflective awareness of one's intentional action, establishes the basis of individual's “practical attunement with the surrounding world of objects and others” (Gallese and Sinigaglia, 2010, p. 753).

To date, these phenomenological hypotheses have had a positive validation thanks to neuroscientific findings that highlighted the crucial role of the motor system in action perception. Beginning in the 1990s, experimental studies on non-human primates evidenced that the motor system is not limited only to the control and production of movements, but that it is also involved in cognitive functions. For instance, it was shown that monkey's ventral premotor neurons encode goal-related motor acts regardless of the effector and the sequence of movements required to accomplish the goal (Rochat et al., 2010)and that the same motor area responds differently when an observed action occurs in the peri- or extra- personal space (Caggiano et al., 2009). Accordingly, these findings indicate that the monkey's motor system is organized not in terms of movements, but rather in terms of goal-directed motor acts. More recently, several experiments provided similar evidence for the human motor system as well. In particular, Cattaneo et al. (2010) demonstrated that the observation of a tool movement activates the cortical motor representation of the hand movements involved in the observed motor behavior, and that the observation of the tool goal-related motor act activates a cortical representation of the observed motor goal. Hence, goal-directed motor acts could be considered as the nuclear building blocks around which action is produced, perceived, and understood through embodied simulation mechanisms (Gallese and Sinigaglia, 2010).

In fact, with the discovery of mirror neurons (Rizzolatti and Fogassi, 2014), motor neurons were shown to be able to code peripersonal space and transform object affordances into potential motor acts (Rizzolatti et al., 1997). Seeing a manipulable object (e.g., a tool) selectively recruits the same motor resources typically employed during the planning and execution of actions targeting the same objects. Several studies performed on human and non-human primates demonstrated that the same neuronal populations in the premotor and posterior parietal cortex are selectively activated both when grasping an object and perceiving it (Gallese and Sinigaglia, 2013). It is also worth noting that during object perception, the recruitment of grasping motor representations can be affected by the same spatial constraints that govern the execution of grasping actions. Behavioral studies showed that the ability of an object to afford a suitable grip depends on its actual reachability, even when people do not act upon it, nor intend to do it (Costantini et al., 2010) and that affordability is context dependent (Costantini et al., 2011). Therefore, spatial constraints affect one's reuse of his/her own action representations—a finding corroborated in a study performed with transcranial magnetic stimulation (TMS) (Cardellicchio et al., 2011). In addition, several electroencephalographic (EEG) studies demonstrated that viewing a tool automatically activates its motoric properties, including its affordance as well as the representation of the associated motor interaction. These results show that the functional identity of graspable objects influences the extent to which they are associated with motor representations (Creem-Regehr and Lee, 2005; Proverbio, 2012). Moreover, object familiarity could enhance the activation of action representations and motor plans (Rüther et al., 2014).

Overall, such EEG studies show that the action plan to interact with objects engenders a suppression of the sensorimotor rhythm across motor areas, a peculiar EEG rhythm which was already demonstrated to be sensitive to action goals irrespective of sensory modalities (Vanderwert et al., 2013). In fact, studies reported a mu rhythm de-synchronization during execution and observation of both goal-directed (Muthukumaraswamy et al., 2004) and non-goal directed (Babiloni et al., 1999) hand movements. Hence, the observation of tools, as well as goal and non-goal directed motor action, leads to an extraction of information about potential affordances and this information lies in the neuronal population of motor areas.

## Immersive virtual reality as a tool for neuroscientific investigation of architectural experience

During the last two decades, a growing number of experimental studies in psychology and neuroscience have started to use the Immersive Virtual Reality (IVR) as a tool for investigating human behavior and brain activity during the natural interaction with the external world (Tarr and Warren, 2002; Sanchez-Vives and Slater, 2005; Bohil et al., 2011; Dombeck and Reiser, 2012). In contrast to classical experimental methodologies in which reality is often reduced to text-, graphic- or computer-based abstractions of the real world typically presented through a display monitor, the experimental approaches based on IVR have the capacity to induce an experience where the user is “surrounded by a three-dimensional computer-generated representation, and is able to move around in the virtual world and see it from different angles, to reach into it, grab it, and reshape it” (Rheingold, 1991). In this section we discuss the potential of using IVR tools to investigate perceptual and emotional responses to architectural environments. First, we highlight key aspects of this technology for examining neurophysiological activity and then propose guidelines for VR research of architectural experience based on previously established postulates of the enactive approach.

### Immersion and presence to create reality in virtual scenarios

The term “immersive” in virtual reality derives from the device's ability to induce an high degree of “immersion” within the virtual world, based on the number and range of user's sensory and motor channels connected to the system (Slater, 1999; Sanchez-Vives and Slater, 2005). According to Slater and Wilbur, the concept of Immersion can be defined as description of a technology and indication of “the extent to which the computer displays are capable of delivering an inclusive, extensive, surrounding, and vivid illusion of reality to the senses of a human participant” (Slater and Wilbur, 1997; see Witmer and Singer, 1998, for a different interpretation). Such immersive experience is generated through a combination of different technologies working as a unified system, which delivers visual information changing in real time according to the movement of the user's head and body, as if he/she was in an equivalent physical environment (Slater, 1999; O'Regan and Noë, 2001). In particular, these “immersive” systems deliver stereo images as a function of the head-tracking that allows the user to freely explore and navigate in the virtual environment as well as to interact with the three-dimensional objects if the system is combined with haptic devices.

In this way, the IVR technologies offer the possibility to create sensory environments that can be replicated almost identically to the reality under the full control of the experimenter, as well as to design scenarios and conditions that are too expensive, dangerous, or impossible to create in physical reality. Importantly, by granting the user freedom to explore, move, and act in the environment in a natural way—that is, by establishing the natural sensory-motor interaction between the user and the virtual world—the IVR gains a fundamental advantage reflected in an high level of ecological validity (Gibson, 1969). Accordingly, IVR environments can be considered to be highly controlled and enriched simulations of the real world and therefore, they enable investigation of human behavior and brain processes during a natural interaction with the ambient (Bohil et al., 2011). Evidently, it is precisely for these reasons that IVR technology provides the means to study with considerable accuracy a wide variety of architectural scenarios and the corresponding behavioral and experiential patterns.

**Figure 5:**
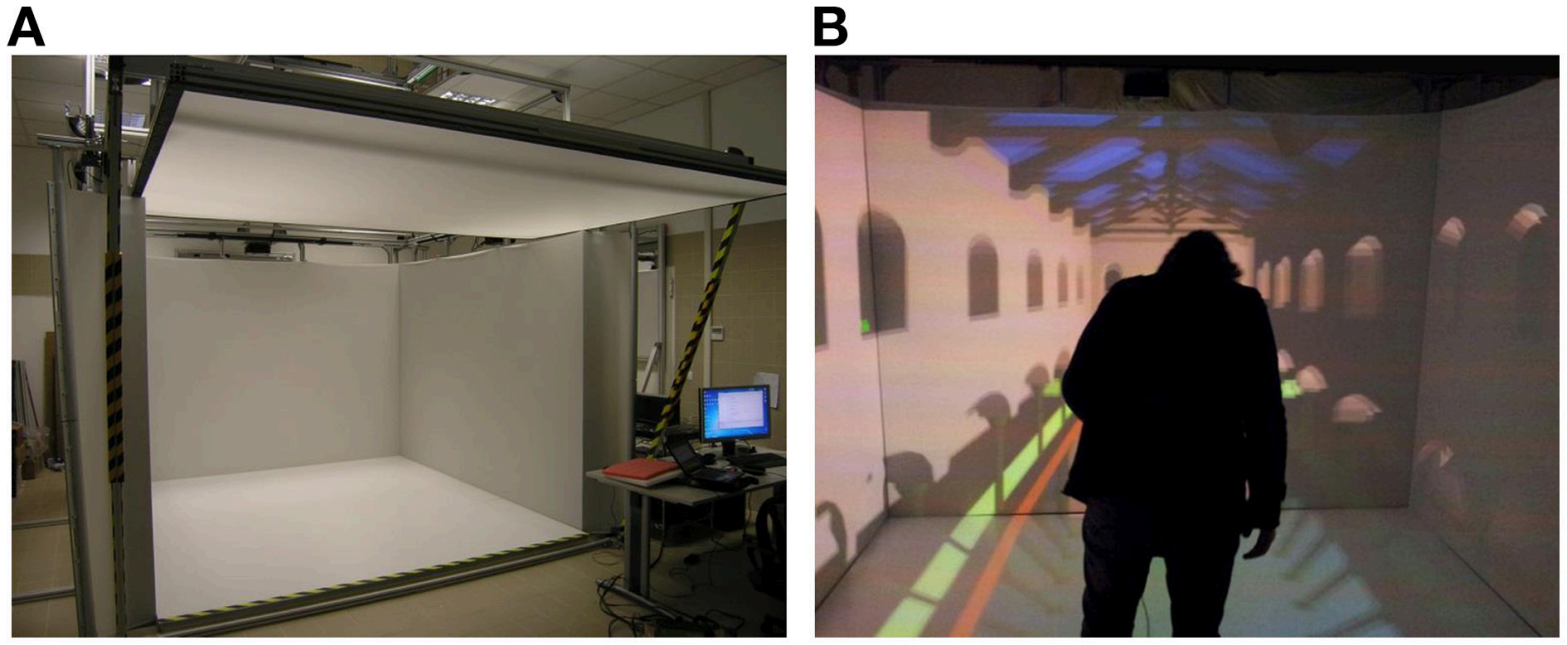
**(A)** Example of a 5 screen Cave system (SCN Lab at IRCCS Santa Lucia Foundation). **(B)** A user in the Cave during the appreciation and interaction with the three-dimensional environment.

The IVR tools currently used are the Head-Mounted Display (HMD)—a wearable immersive device – and the Cave Automatic Virtual Environment (CAVE)—a room-like VR setting (Cruz-Neira et al., 1993; Figure 5). Therefore, in contrast to designing virtual environments on a desktop monitor that generally displays image sequences fixed on the screen and does not allow any natural interaction, such immersive technologies enable to design a virtual world that responds in real time to the movement of the user that thus becomes part of the three-dimensional environment. According to Slater (1999), these sensory-motor contingencies play a crucial role in characterizing a system as immersive.

**Figure 6:**
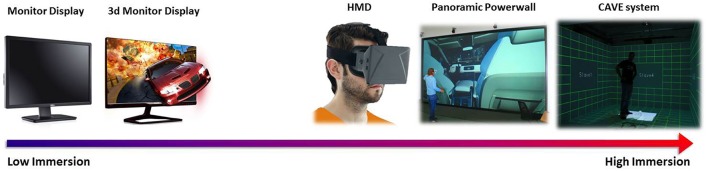
**Schematic representation of the IVR technologies able to elicit the sense of presence based on their level of immersivity**.

A second fundamental value of the IVR for experimental paradigms resides in the evidenced capacity that the exposure to an immersive virtual scenario can elicit in the participants a strong feeling of “being physically present” in the perceived environment (Draper et al., 1998), which represents an important aspect of consciousness (Seth et al., 2012). This capacity is defined conceptually as the sense of presence and it is reflected in a modulation of the psychophysiological and neurophysiological responses which reproduce realistic behavior and physiological reactions as if the subject is physically situated in a real place (Sanchez-Vives and Slater, 2005; Parsons and Rizzo, 2008). Because of its centrality to the research potential of the IVR tools, the sense of presence has been extensively measured by means of different methodologies that are commonly based on: (i) the standardized questionnaire that provides a subjective judgment regarding the user's experience of presence (Freeman et al., 1999); (ii) the measure of behavioral responses elicited by introduction of features into the virtual environment that can cause a bodily response, including the looming response (Held and Durlach, 1992), postural sway (Freeman et al., 2000), after-effects (Welch et al., 1996), and conflicting multi-sensory cues (Slater et al., 1995); (iii) the use of the event-based “breaks in presence” (Slater and Steed, 2000) i.e., the phenomenon during the VE exposure that launches the participant into awareness of the real-world setting of the experience, and therefore, breaks their presence in the VE; and the measure (iv) of person's physiological activities, e.g., Heart Rate (HR) and Skin Conductance (SC) (Meehan et al., 2002, 2005; Slater, 2009; Peperkorn et al., 2014; Vecchiato et al., 2015b); and (v) the electroencephalographic (EEG) responses elicited by exposure to a VE (Kober et al., 2012; Slobounov et al., 2015; Vecchiato et al., 2015a,b) that provide objective measures of person's body and brain activities respectively. In Figure 6 we summarize the IVR devices according to the different degrees of presence they elicit.

### How to investigate embodiment, motivation, and affordances in architectural context with IVR

By exemplifying how sensorimotor and interoceptive activity can be measured in different experimental IVR setups, including a few architecture-specific studies, we offer several indications how the enactive approach to architectural experience could be tested through IVR paradigms.

#### The sense of presence modulates emotional response and interoceptive activity

The investigation of person's internal states during a VR experience—measured by questionnaire and physiological activities—covers a wide range of works that goes from studying the effects of simple perceptual factors in the VE (e.g., the shadow of the user's virtual body reflected on the floor, Slater, 2009) to the emotional responses and their correlations with the sense of presence, (Meehan et al., 2002, 2005; Peperkorn et al., 2015). This issue is of particular relevance because the tight relationship between elicited emotional response and presence during virtual scenario exposure has been marked as an indicator of the accuracy and validity of the simulated experience. In fact, Meehan et al. (2002) showed that the degree to which a virtual environment seems real results in a high similarity between the physiological responses evoked in the VR and those in the equivalent real environment. By means of a HMD, they tested this hypothesis by introducing in the VR paradigm a stressing event—in this case a stressful virtual height situation elicited by a virtual hole on the floor of the pit room—in order to trigger a physiological response. In this way, authors compared participants' physiological reactions (i.e., Heart Rate, Skin Conductance, and Skin Temperature) during the exposure to a stressful vs. non-stressful virtual scenario. As a general result, they found that participant's anxiety responses (HR and SC) were significantly higher during the stressful event and that these measures correlate with the level of presence (as assessed with a questionnaire). Thus, this finding implies that high level of presence corresponds to higher physiological activities (HR and SC) in response to an external threat (result also found in Meehan et al., 2005). Similar evidence about the relationship between physiological activity and sense of presence was reported by Vecchiato et al. (2015b), where instead of a stressful event participants were exposed to three different architectural interiors. For all presented scenarios, authors found that high and low levels of presence were respectively reflected in higher and lower HR responses, recorded during simple observation of virtual environments in a CAVE.

Overall, even though the perceptual factors affecting the relationship between presence and emotion are still not fully understood (Diemer et al., 2015), the aforementioned evidence highlight three important information: (i) high level of presence corresponds to higher physiological activities (HR and SC) (Meehan et al., 2002, 2005); (ii) physiological responses (HR and SC) can serve as objective and reliable measures of presence (Meehan et al., 2005); and (iii) presence is a necessary mediator that allows real emotions to be activated by a virtual environment (Parsons and Rizzo, 2008). Such evidence also comes in support of the recent efforts to define the enactive concept of presence and emotion in the context of virtual reality, an aspect which has thus far received little to no attention (Willans et al., 2015). In particular, Willans and colleagues endorse the enactive approach to emotion as developed by Colombetti and Thompson (Colombetti and Thompson, 2008; Colombetti, 2014) and formulate the enactive approach to presence postulating that: (i) the sense of presence emerges from meaningful and self-sustaining actions resulting from dynamic reciprocal interaction between the organism and the environment, and not as a simulated world in the head; (ii) the sense of presence occurs where the emotional episode takes place and it is formed in the same way through dynamic self-organizing patterns; and (iii) presence is located where the phenomenological self is emplaced, where the self exists in the symbiosis between the natural and synthetic body (Willans et al., 2015). While such paper indicates a potentially fruitful direction for theoretical conception of presence and emotion for VR on the basis of the enactive approach, more detailed proposal is beyond the scope of the present work.

Additional knowledge comes from the studies particularly focused on the emotional aspect elicited by a VR experience. As described in Diemer et al. (2015), these studies used the VR as a medium for investigating emotional responses, e.g., sadness, joy, relaxation (Baños et al., 2004, 2008), anxiety (Juan and Pérez, 2009), arousal (Freeman et al., 2005), fear (Diemer et al., 2015; Peperkorn et al., 2015), and their relationship with presence. In the work of Riva et al. (2007), authors used a HMD to immerge participants in a virtual open space green park that was represented in three different fashions (relaxing, anxious, and neutral). The results demonstrated that the interaction with “anxious” and “relaxing” virtual environments produced feelings of anxiety and relaxation respectively, and that the emotional states were influenced by the level of presence. Similarly, Baños et al. (2004) used two virtual environments representing a park with a neutral and gloomy atmosphere, which were presented by means of HMD, big screen, and monitor display. Their analysis showed that the emotional valence of the VE affects the emotional state of the user—thus extending previous observations indicating that the immersion and affective content play a role in eliciting the sense of presence (Baños et al., 2004, 2008).

Interestingly, recent study by Fich et al. (2014) confirmed this relationship by examining if and how particular features of architectural design influence the participants' stress response. By using a Trier Social Stress Test in a CAVE setup, participants performed stressful tasks (e.g., giving a presentation in front of a committee) in two virtual scenarios, a room with large openings and a closed room, while monitoring physiological activity in terms of heart rate variability and cortisol level in saliva samples. What they found is that participants exhibited greater reactivity to stress when doing the tasks in the enclosed room, which was especially manifested in the cortisol reactivity controlled by the HPA-axis stressor system, a response that could be associated with whether the subject perceives the situation as controllable. Accordingly, because the HPA-axis is partially governed by the feedback from hippocampus, a known brain area involved in encoding characteristics of space boundaries, the authors suggested that the greater stress reaction to enclosed space could be related to the limited possibility to move and escape. Lastly, it is also worth noting that preliminary testing returned the evidence of differences in emotional responses between architects and non-experts when observing distinct spatial geometries in an immersive virtual reality, which is an issue that will examined in more details in future studies (Shemesh et al., 2015).

#### Neuroelectrical correlates of sensorimotor activity and embodiment

Recent neuroscientific studies investigated the experience in VR looking directly at the brain activity by monitoring EEG signals (Kober et al., 2012; Slobounov et al., 2015; Vecchiato et al., 2015a,b). For instance, in Vecchiato et al. (2015b), authors recorded EEG activity in individuals freely observing three virtual architectural interiors—a residential room furnished as empty, modern, and cutting-edge—presented through a CAVE. The EEG analysis showed that the sense of presence (measured by questionnaire) was reflected in the activation of the frontal-midline theta over a brain network that includes frontal, orbitofrontal, and left temporal areas, with the activity of these regions particularly increased in the state of “high presence.” This finding is supported by two studies that used a spatial navigation tasks in virtual reality. Indeed, Slobounov et al. (2015) examined the effect of fully immersive 3D stereoscopic presentations and less immersive 2D VR environments on brain functions and behavioral outcomes. Results showed that during the state of presence in immersive 3D scenario, subjects reported an enhancement of frontal midline theta (FM-theta) correlated with the success rate in a spatial navigation task. Similarly, Kober et al. (2012) compared the subjective feeling of presence and its underlying cortical correlates in two different immersive VR systems (high vs. low immersivity). They observed that a higher level of experienced presence (recorded in the high immersivity condition) was reflected in a stronger parietal brain activation compared to low immersivity condition. These observations suggest that the sense of presence could elicit mechanisms underlying sensorimotor integration as well as cerebral networks regulating focused attention (Vecchiato et al., 2015b). In addition, the authors noted that FM-theta was elicited during the visuospatial exploration of environments judged as more familiar, comfortable, and pleasant. These findings may reflect recruitment of theta oscillations in focused attention and positive emotional experience mechanisms associated with the exploration of VEs. Therefore, the recognition of familiar features in the environment, as well as the perception of comfort, could activate those cerebral circuits involved in internalized attention, relaxation and hence favor sensorimotor integration in space (Vecchiato et al., 2015b).

In parallel, various researchers used virtual reality-based paradigms to investigate neural mechanisms underpinning bodily-awareness and more generally, the phenomenon of embodiment. For example, bodily-awareness has been examined by using IVR to substitute the real body of participants by manipulating its shape and limb symmetry (Kilteni et al., 2012), visual appearances (Tieri et al., 2015a,b), skin colors (Peck et al., 2013), and the perspective point of view by which the body is perceived (Slater et al., 2010; Pavone et al., 2016) and its application in neurorehabilitation (Tidoni et al., 2015). In addition, a recent study by Pasqualini et al. (2013) explored how architectural interiors—a large and narrow space respectively—modulate bodily self-consciousness on account of visuo-tactile mechanisms. By using a video-based setup, involving a virtual body and a HMD, they discovered that while self-identification is independent of the room size, the sidewalls of the narrow space induced weak feelings of illusory touch in the participants. Accordingly, they concluded that the experience of architecture from a first-person, i.e., embodied perspective can be related to the induced changes of bodily self-awareness, by being a result of projection of bodily space and self-identification with the spatial void of the virtual interior.

Taken together, these findings highlight that it is possible to measure EEG correlates of architectural perception involving the cerebral circuits of sensorimotor integration, spatial navigation, and embodiment.

### Limitations and future directions

Finally, due to relative novelty of applying virtual reality paradigms for the purposes of architecture research, some limitations and guidelines for future efforts should be outlined. Primary limitation is concerned with the lack of exhaustive studies on the extent to which user's experiences are analogous in virtual and real environments. Indeed, a recent study with desktop VE showed that while quantitative data returned few statistically significant differences between ratings of the real and virtual building, analyses based on qualitative data revealed differences concerned with the atmosphere of architectural space (Kuliga et al., 2015). Thus, while the VR technology confirmed its potential to become a valuable research tool, a similar study is needed to examine the degree of similarity and differences in the case of more immersive virtual reality tools and experience of the real environments. On the other hand, most of the aforementioned studies rely on rating scales and adjective-based evaluation questionnaires for obtaining participants' subjective judgments. However, because of the nature of enactive approach which is closely related to Varela's proposal for neurophenomenology, it is possible to hypothesize that future studies could include such phenomenologically-based techniques for obtaining first-person experiential reports in addition to traditional cognitive science methods (Bockelman et al., 2013). In fact, based on the described phenomenological legacy in architectural literature and recent efforts to investigate the experience of awe and wonder in VR by applying neurophenomenological method (Reinerman-Jones et al., 2013), it is a plausible direction to embrace in order to develop the closest possible neuroscientific interpretation of architectural experience.

In regard to the hypothesis that the experience of architectural space is directly structured on account of the interactive interdependency between our embodiment, affective states, and perceived spatial affordances, a recent study could offer a direction for developing appropriate IVR paradigms. Meagher and Marsh (2015) conducted five experiments in virtual reality (HMD) and a laboratory with an equal real-life setting with the intention to test how impressions of spaciousness are influenced by behavioral opportunities offered by the environment. Based on participants' spatial judgments (measured by a questionnaire) they investigated two aspects: first, the “*environmental salience hypothesis*” according to which the design of the room (here involving furniture arrangement and room size) can invite and guide a perceiver to engage (or not) in certain activities; and second, the “*behavioral primacy hypothesis*” suggesting that user's impressions of space are based on the room's suitability for the primed activity and motivations of the perceiver. Overall results showed that there exists a strong dependency between the impressions of spaciousness and the perceived possibilities for the participant to act in the space and that the room (virtual and real) was judged as accommodating or inhibiting behavior, especially when previously primed for a particular activity.

## Conclusion

From the enactivist perspective, the way people perceptually experience the world, including architectural spaces, is governed by the dynamic sensorimotor activity of the human organism as a whole and is thereby influenced by the particular conditions of man's embodiment. Accordingly, it can be argued that we engage with architecture through embodied action and that our experience of architecture is constituted by the complex patterns of sensorimotor activity. What is thus suggested is that users are not mere disembodied observers of spaces—instead, the value and meaning of an architectural environment originates in the architecture-body interaction. Here, the proposed enactive framework provides an interpretation of embodiment which makes the body necessary for experience of architecture and emphasizes the intrinsic connection between architecture and human mind/body through action. Moreover, following the sensorimotor theory of perception and the enactive conception of perception as anticipatory and action-oriented we hypothesize that architecture is understood and perceived by providing (designed) affordances as possibilities for action. In fact, affordances can exist and be understood perceptually precisely because body schema connects perception and bodily capacity to act, and thus this functional mechanism can be defined as a communicative point in our engagement with architectural space and understanding of design intentions. Body schema enables an architectural subject to move through the spatial setting in a consciously effortless manner and over time to establish habitual patterns of use. Finally, we argue that an experimental approach based on IVR can be beneficial to investigate complex human perceptions underlying the experience of architectural environments and thus help architects design environments better suited to users' changing needs.

## Author contributions

AJ conceptualized the research. AJ, GV, and GT developed the research and wrote the paper. FDM and FB supervised the research and contributed to revision of the manuscript. All authors provided final approval of the version submitted for review and publication.

### Conflict of interest statement

The authors declare that the research was conducted in the absence of any commercial or financial relationships that could be construed as a potential conflict of interest.

## Footnotes

^1^The architectural theory and methods of space syntax (Hillier and Hanson, 1984) emerged as an attempt to understand the relationship between space and society, emphasizing the co-dependency of (pedestrian) movement patterns and spatial configuration. Space syntax methodology of structural analysis of space is primarily aimed at quantifying the spatial relations in terms of visual accessibility (or vistas available to users) and the connectivity of its parts. To date, collaborations between space syntax researchers and cognitive scientists have yielded compelling results in the study area of wayfinding and navigation in the built environment (Montello, 2007; Dalton et al., 2012).

^2^In Pallasmaa's words, architectural images are “promises and invitations: the floor is an invitation to stand up (…), the door invites us to enter and pass through (…), the staircase to ascend and descend” (Pallasmaa, 2011, pp. 123–124). All these inherent suggestions of action contained in architecture at the time of the encounter inevitably result in a bodily reaction, which is thus “an inseparable aspect of experience of architecture” (Pallasmaa, 2005, p. 63).
